# Comparative highlights on MERS-CoV, SARS-CoV-1, SARS-CoV-2, and NEO-CoV

**DOI:** 10.17179/excli2022-5355

**Published:** 2022-09-29

**Authors:** Rajat Goyal, Rupesh K. Gautam, Hitesh Chopra, Ankit Kumar Dubey, Rajeev K. Singla, Rehab A. Rayan, Mohammad Amjad Kamal

**Affiliations:** 1 MM College of Pharmacy, Maharishi Markandeshwar (Deemed to be University), Mullana-Ambala, Haryana, India; 2 MM School of Pharmacy, Maharishi Markandeshwar University, Sadopur-Ambala, India; 3 Department of Pharmacology, Indore Institute of Pharmacy, Rau, Indore, India-453331; 4 Chitkara College of Pharmacy, Chitkara University, Punjab, India-140401; 5 iGlobal Research and Publishing Foundation, New Delhi, India; 6 Institutes for Systems Genetics, Frontiers Science Center for Disease-Related Molecular Network, West China Hospital, Sichuan University, Chengdu 610041, Sichuan, China; 7 School of Pharmaceutical Sciences, Lovely Professional University, Phagwara, Punjab-144411, India; 8 Department of Epidemiology, High Institute of Public Health, Alexandria University, 5422031, Egypt; 9 King Fahd Medical Research Center, King Abdulaziz University, Saudi Arabia; 10 Department of Pharmacy, Faculty of Allied Health Sciences, Daffodil International University, Bangladesh; 11 Enzymoics, 7 Peterlee Place, Hebersham NSW 2770; Novel Global Community Educational Foundation, Australia

**Keywords:** coronavirus, COVID, SARS-CoV-1, SARS-CoV-2, MERS-CoV, Neo-CoV, respiratory syndrome

## Abstract

The severe acute respiratory syndrome (SARS-CoV, now SARS-CoV-1), middle east respiratory syndrome (MERS-CoV), Neo-CoV, and 2019 novel coronavirus (SARS-CoV-2/COVID-19) are the most notable coronaviruses, infecting the number of people worldwide by targeting the respiratory system. All these viruses are of zoonotic origin, predominantly from bats which are one of the natural reservoir hosts for coronaviruses. Thus, the major goal of our review article is to compare and contrast the characteristics and attributes of these coronaviruses. The SARS-CoV-1, MERS-CoV, and COVID-19 have many viral similarities due to their classification, they are not genetically related. COVID-19 shares approximately 79 % of its genome with SARS-CoV-1 and about 50 % with MERS-CoV. The shared receptor protein, ACE2 exhibit the most striking genetic similarities between SARS-CoV-1 and SARS-CoV-2. SARS-CoV primarily replicates in the epithelial cells of the respiratory system, but it may also affect macrophages, monocytes, activated T cells, and dendritic cells. MERS-CoV not only infects and replicates inside the epithelial and immune cells, but it may lyse them too, which is one of the common reasons for MERS's higher mortality rate. The details of infections caused by SARS-CoV-2 and lytic replication mechanisms in host cells are currently mysterious. In this review article, we will discuss the comparative highlights of SARS-CoV-1, MERS-CoV, SARS-CoV-2, and Neo-CoV, concerning their structural features, morphological characteristics, sources of virus origin and their evolutionary transitions, infection mechanism, computational study approaches, pathogenesis and their severity towards several diseases, possible therapeutic approaches, and preventive measures.

## Introduction

The most common pathogens that primarily affect the human respiratory system are the influenza A virus, severe acute respiratory syndrome coronavirus (SARS-CoV-1), middle east respiratory syndrome coronavirus (MERS-CoV), and 2019 novel coronavirus (SARS-CoV-2). Diseases allied to their infections range from mild respiratory sickness to acute pneumonia and in some cases, failure of the respiratory system also (Abdelrahman et al., 2020[1]). Coronaviruses are the largest enveloped positive RNA (+RNA) viruses that belong to the family 'Coronaviridae', and order 'Nidovirales'. They can acclimatize to newer environments via mutation and recombination and they are programmed to amend the tissue tropism and host range. MERS is a viral infection that may lead to acute respiratory infections though the spectrum contrasts, with few asymptomatic cases and others leading to a potentially lethal disease with a higher mortality rate. The threats posed by the SARS- and MERS-related coronaviruses to human well-being are continual and long-standing. Nevertheless, we are missing detailed knowledge about the pathogenesis of these coronaviruses which makes the treatment difficult to determine, whether in the drug selection or vaccine development (Nascimento Junior et al., 2020[104]).

In addition to SARS-CoV-2 (COVID-19), there are six other human-infecting coronaviruses. Amongst them, SARS-CoV-1 instigated over 8000 infections and around 900 deaths in thirty-two countries from 2002 to 2004; MERS-CoV triggered the epidemics in around twenty countries in 2012. As of January 2020, over 2500 human infections had been reported, with around 866 deaths. The high mortality rate of the infections induced via MERS-CoV, and SARS-CoV, as well as the enduring pandemic triggered by SARS-CoV-2, indicates that coronaviruses are amongst the most lethal viruses in human health (Cai et al., 2021[15]). SARS-CoV and MERS-CoV are considered to be highly infective and likely to be spread from bats to palm civets, dromedary camels and human beings finally (Wu et al., 2020[162]).

SARS-CoV begins as a flu-like illness that progresses to pneumonia, failure of the respiratory system, and death in a few cases. The mortality rate due to SARS-CoV is much higher than influenza or other common respiratory system infections. The various symptoms of SARS-CoV infections are headache, diarrhea, shivering, fever, malaise, and myalgia (Ezhilan et al., 2021[43]). SARS-CoV-2 is an extremely contagious and infective coronavirus that emerged in late 2019 and instigated a pandemic of acute respiratory illnesses termed as 'coronavirus disease-2019' (i.e., COVID-19), that targets the health of human beings and their safety (Hu et al., 2021[55]). The patients suffering from SARS-CoV-2 viruses have chronic respiratory illnesses. The symptoms caused by SARS-CoV-2 comprise of breath shortness, sore throat, runny nose, nausea, pain, diarrhea, and aches. On the other hand, MERS-CoV is a single-stranded RNA virus that frequently binds to the DPP4 receptor and enters the host cell. 

**Figure 1:**
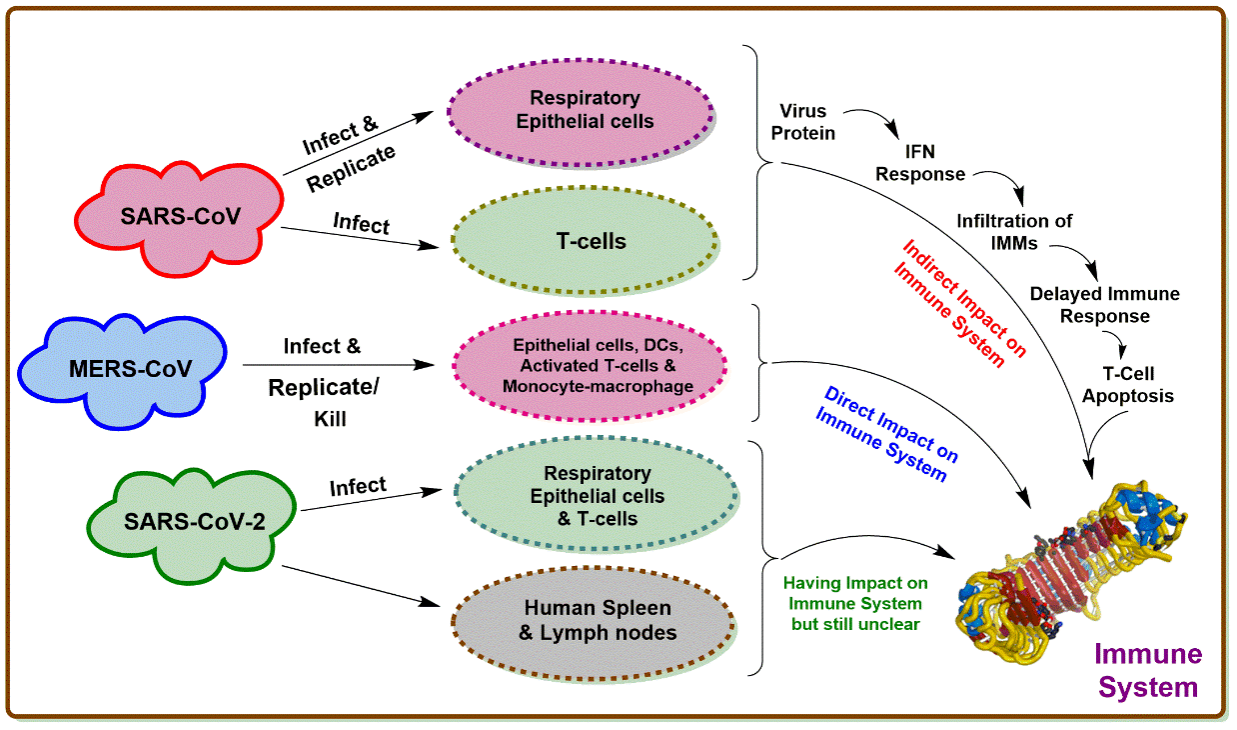
The difference between host cells among SARS-CoV-1, SARS-CoV-2, and MERS-CoV, and their impact on the immune system

The first case of MERS coronavirus was observed in 2012 in Saudi Arabia by a patient suffering from a flu-like respiratory illness. The most communal symptoms of MERS coronavirus include cough, fever, breath shortness, diarrhea, pneumonia, and GIT-associated illnesses. There is not any effective vaccination or treatment therapy for MERS-CoV, so far. Lopinavir, interferon, and convalescent plasma are utilized to treat MERS-CoV patients (Ezhilan et al., 2021[43]). The difference between the host cells among SARS-CoV-1, SARS-CoV-2, and MERS-CoV, and their impact on the immune system (Liang et al., 2020[87]) is summarized in Figure 1(Fig. 1).

## Structural Features and Morphological Characteristics of Viruses

**Figure 2:**
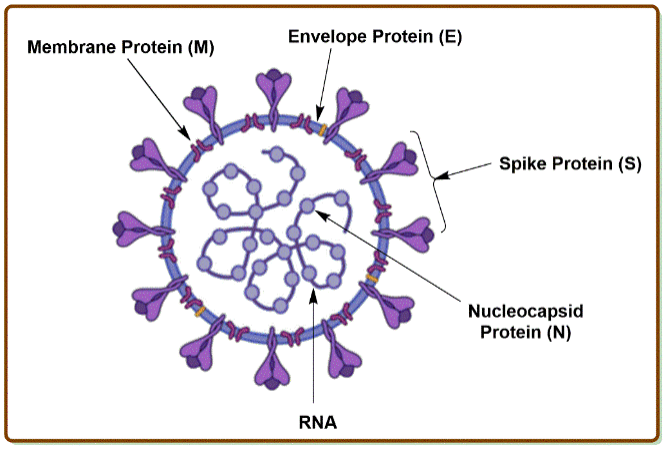
The structure of coronavirus

Coronaviruses are a large and most diversified group of RNA viruses, consisting of a positive-sense RNA genome, that acts directly as a messenger RNA during the viral replication privileged to the host cell. They are characterized as spherical-shaped particles that acquire a viral envelope from the host cytoplasmic membrane, which is obligatory for the host cell attachment and leads to the succeeding replication steps. Coronaviruses cannot replicate if there is a disintegration of the viral envelope. The spike (S) glycoproteins on the surface of viral envelopes are critical for the attachment to host cell receptors. Coronavirus consists of a nucleocapsid (N) protein inside the viral envelope that is essential for the processes of replication, pathogenesis, infection, virulence, and dissemination. This N-protein is helically symmetric and is surrounded by RNA coils. The viral RNA in N-protein encodes all the heredity information for the mechanism of replication, host cell docking, viral encoded protein synthesis, mutilation, and the development of diseases (Kannan et al., 2020[65]), as described in Figure 2(Fig. 2).

Coronaviruses are categorized into four classes such as alpha-coronavirus (α-CoV), beta-coronavirus (β-CoV), gamma-coronavirus (γ-CoV), and delta-coronavirus (δ-CoV). Among them, both α-CoV and β-CoV infect the mammals, whereasγ-CoV infects the avian species, and δ-CoV infects both the mammalian and avian species. Human coronavirus-NL63 (HCoV-NL63), porcine transmissible gastroenteritis coronavirus (TGEV), and porcine respiratory coronavirus (PRCV) are examples of α-CoV. MERS-CoV, SARS-CoV, mouse hepatitis coronavirus (MHV), bat coronavirus HKU4, bovine coronavirus (BCoV), and human coronavirus OC43 are examples of β-CoV, while, avian infectious bronchitis coronavirus (IBV) and porcine delta-coronavirus (PdCV) are examples of γ-CoV and δ-CoV, respectively (Li et al., 2016[82]).

### SARS-CoV 

Coronavirus genomes are the largest among RNA viruses, the coronavirus family is named due to the large spike protein molecules found on the virus surface, which give the virions a crown-like shape (Pellett et al., 2014[114]). SARS-CoV is a positive-stranded RNA virus that has been described as a giant, enveloped, and positive-stranded RNA virus with a genome of 29,727 nucleotides (around 30 kb), about 41 % of which are cytosine or guanine. This virus's genomic body has the original gene order of 5'-replicase (rep), which accounts for two-thirds of the genome and comprises the large genes ORF1a and ORF1b. Furthermore, four open reading frames (ORFs) downstream of the rep gene encode the 3' structural spike (S), envelope (E), membrane (M), and nucleocapsid (N) proteins. Rep gene products are derived from the genomic RNA, whereas the remaining viral proteins are derived from the sub-genomic mRNAs (Tan et al., 2006[146]).

### MERS-CoV 

Although MERS-CoV belongs to the same family, order, and genus as that of SARS-CoV, it was the first beta-coronavirus lineage C member to be recognized as a “novel coronavirus” with a genomic size of 30,119 nucleotides. The genome of MERS-CoV encodes ten proteins, which comprise two replicase polyproteins (i.e., ORF1ab and ORF1a), four structural proteins (i.e., S, E, M, and N), and four non-structural proteins (Chung et al., 2019[33]).

### SARS-CoV-2 

As SARS-CoV-2 is also from the similar family and genus as SARS-CoV-1 and MERS-CoV, the genomic analysis revealed a higher degree of similarity between SARS-CoV-1 and SARS-CoV-2. Initially, the International Committee on Taxonomy of Viruses' Coronaviridae Study Group recognized this virus as a sister clade to the prototype human and bat severe acute respiratory syndrome coronaviruses (SARS-CoVs). Later, it was labelled as SARS-CoV-2 (Grifoni et al., 2020[47]).

The genomic analysis of SARS-CoV-2 demonstrated that the genome comprises of six major ORFs and shares less than 80 % nucleotide sequence identity as that of SARS-CoV. Though, the seven conserved replicase domains in amino acid sequence of the ORF1ab share 94.4 % identity with SARS-CoV. The SARS-CoV-2 genome is also highly similar to that of the bat coronavirus (Bat CoV RaTG13), having a sequence identity of 96.2 %. Moreover, the receptor-binding spike protein is 93.1 % identical to Bat CoV RaTG13 (Chen et al., 2020[25]). Meanwhile, significant differences in S-gene sequence of SARS-CoV-2 were observed in comparison to SARS-CoV, including three short insertions in the N-terminal domain, changes in 4 out of 5 crucial residues in the receptor-binding motif, and the existence of an unexpected furin cleavage site at the S1/S2 boundary of the spike glycoproteins of SARS-CoV-2. This inset distinguishes the SARS-CoV-2 from SARS-CoV-1, and other SARS-related coronaviruses (Andersen et al., 2020[6]).

### Neo-CoV

MERS-CoV and Neo-CoV shared the critical genomic architecture details. At the nucleotide level, 85 % of the Neo-CoV genome was found to be similar to the MERS-CoV. Both MERS-CoV and Neo-CoV belonged to the same species of virus, according to the taxonomic criteria. The existence of an inherently divergent S1-subunit inside the Neo-CoV S gene suggested that the intra-spike recombination proceedings played a vital role in MERS-CoV emergence. Neo-CoV creates a sister taxon of the MERS coronavirus, which places the root of MERS-CoV amongst a recently pronounced virus from African camels and all additional viruses. These studies suggest that camels have a higher level of viral diversity than human beings (Corman et al., 2014[35]).

## Sources of Virus’s Origin and their Evolutionary Transitions

The RNA virus field has progressed quickly, especially the family of viruses known as 'Coronaviridae', which are members of the 'Nidovirales' order and have genomes of less than 30 kilobases (Kb) (Sevajol et al., 2014[134]). These deadly coronaviruses (i.e., SARS-CoV-1, MERS-CoV, and SARS-CoV-2) may transcend species borders and lead to serious sickness and death in humans because of their rapid genetic evolution. In general, viral genomes encode proteins that are essential for three major purposes, including structural proteins, transcription and replication proteins, and proteins that allow the virus to infect the host cells.

A non-structured protein, RNA-dependent polymerase, which aids in the replication and transcription of viral RNA, is synthesized from the open reading fragments (ORFs)-1a and 1b of viral genomes (Mizutani et al., 2000[98]; Weidmann et al., 2004[158]). Two-thirds of the virus's genome of these proteins are involved in the viral replication and in the creation of sub-genomic mRNA, which codes for the various structural and auxiliary proteins (Rota et al., 2003[127]; Snijder et al., 2003[138]). Compared to ORF 1a's (proteins 1-11), which encode structural proteins, ORF 1b's non-structural proteins (proteins 12-16) are far less abundant (Snijder et al., 2003[138]). Viral propagation is facilitated by the following non-structural proteins: Several proteins are involved in the viral RNA replication and transcription, including protein-12 (a 3′-5′ exonuclease having clear proof-reading property), protein-14 (a 3′-5′ exonuclease having clear proof-reading property), protein-15 (an endo-ribonuclease having unclear endo-ribonuclease property), proteins-7 and 8, which act as activators of polymerase co-factors, and protein-10, which acts as a 2′ O-methyltransferase (Minskaia et al., 2006;[97] Ivanov et al., 2004[60]; Egloff et al., 2004[42]; Su et al., 2006[142]). The S, M, E, and N regions of protein encode spike glycoproteins, membrane proteins, envelop proteins, and nucleocapsids, respectively. Recombinant lethal virus types may be generated by mutating or inserting/depleting the ORFs that are scattered across these genes.

SARS-CoV is a zoonotic virus, that has crossed species boundaries from bats to people through civet intermediate hosts (i.e., Bat-CoVs) like other human coronaviruses (HCoVs), according to the present data. The genomic length of the SARS-CoV virus was shorter than that of other HCoV strains, making it a more genetically complex virus than others. The pathogenicity of these organisms to host cells was revealed to have specific purposes. In the ORF 12, ORF 9, ORF 10, and ORF 14 areas of SARS-CoV, many recombinant portions resulted via horizontal transmission or recombination. It has been reported that the SARS-enhanced CoV's pathogenicity is due to a 29-nucleotide deletion of ORF 8a and ORF 8b in ORF 8 (Oostra et al., 2007[108]). Both animal and human isolates shared a single ORF 8 gene, however, only 29-nucleotide deletion strains were detected in human infection throughout the middle and late phases. A successful strain transmission was made possible by the 29-nucleotide deletion (Lau et al., 2005[77]; Guan et al., 2003[48]). 

According to researchers, the S protein mutation has been linked to species transfer and adaptation to human hosts. As a receptor, the S protein must have a receptor-binding domain to recognize the angiotensin-converting enzyme 2 (ACE2). There are several other proteins encoded by the S-region genes that help in the virus' entry into the host cell, including the following: cytoplasmic domain, fusion protein, heptad repeats, receptor-binding site, receptor-binding motif, transmembrane protein (TM), signal peptide (SP). Overexpression of the main proteins 3b and 7a aids in cell death and cell cycle arrest (Yuan et al., 2005[176]; Tan et al., 2004[145]; Yuan et al., 2006[177]). Proteins 3b and 6 (interferon antagonists) have a significant function as well (Pewe et al., 2005[116]). It was fascinating to hear that the hemagglutinin-esterase protein is missing from SARS-CoV, which is a critical virulence component in other HCoVs. A gene producing the hemagglutinin-esterase protein may be present even if there is no indication of widespread recombination in the surrounding region (Su et al., 2016[144]). They have all developed through time so that the virus may adhere and invade, enabling it to become pathogenic.

As the evolutionary host, bats gave rise to the MERS-CoV, the sixth iteration of the coronavirus, which went on to infect humans through jumping species, principally camels (specifically, *Camelus dromedarius*) (Anthony et al., 2017[8]; Memish et al., 2013[96]). A 29Kb base pair single-stranded RNA genome (ssRNA) was used to encode the ORFs 1a and B, as well as the structural proteins- E, M, and, the surface glycoprotein-S (Mackay et al., 2015[91]). To facilitate membrane fusion, the S protein of the MERS-CoV virus is broken into two pieces when it interacts with DPP4 receptors in human cells (Raj et al., 2013[123]). Receptor binding domain S1 (C-terminal 240 residues) includes a core and an outer sub-domain to recognize DPP4 receptors (Moreno et al., 2017)[101]. Pre-hairpin intermediate S2 protein conformational changes are initiated by virions' fusion with cell receptors, and the RBD region is a key player here. There are many pre-hairpin structures in the host cell membrane where the hydrophobic HR1 subunit inserts pre-fusion peptides. Six-helix bundle structures are created by refolding the intermediate with HR2, which tugs the host cell membrane even closer to the virion envelope, assisting in the integration of the viral envelope. 

The MERS-CoV isolates from the patients of South Korea include twenty-nine nucleotide inclusions and twelve amino acid changes that are unique from those seen in other MERS-CoV strain genomes, as previously noted (Kandeil et al., 2016[63]). Another study found that despite 99 percent nucleotide homology between the eight South Korean strains of MERS and the Riyadh strain, thirteen different nucleotides were found in 24 to 27 nucleotide positions across the genome, including six variations in the ORF1ab gene, five in the S gene, and one in each ORF 4aa and a and a, indicating micro-evolution during an outbreak (Seong et al., 2016[132]).

Although structural proteins from human SARS-CoV have yet to be discovered in a bat progenitor, the ORF1ab gene is the most closely connected to human SARS-related coronaviruses. For example, in contrast to the human SARS-CoV virus, the bat SARS-CoV virus has a small deletion in its receptor-binding domain (RBD) that prevents it from interfacing with ACE2 protein (Li, 2013[81]; Ren et al., 2008[124]). When the human SARS-CoV homolog replaced the spike gene in a reverse genetically created bat SARS-related coronavirus, it was exclusively infectious in the mice and cell culture (Becker et al., 2008[13]). RBD of European rhinolophid bats and SARS-coronaviruses were more closely connected to the RBD of human SARS-CoV than the RBD of Chinese bat viruses. This suggests that recombination may have been involved in the creation of the human deadly virus. Although just five rhinolophid bat species have been investigated for SARS-related coronavirus sequences, at least 12 of the 19 species have been tested for the virus (Yuan et al., 2010[175]; Yang et al., 2013[169]; Poon et al., 2005[118]; Li et al., 2005[85]). Research on the species of Rhinolophus in Europe, Asia, and Africa, might deliver information about the origins of human SARS-CoV. To distinguish them from those associated with SARS, the Hipposideros β-CoV discovered in Asia and Africa need their section. These viruses may be identified using any of the two taxonomic methods mentioned above. The genomic characteristics and phylogenetic position of the unclassified Hipposideros β-CoV are different from those of SARS-related coronaviruses. For example, each Hipposideros CoV has a different viral 3'-genome end, as well as additional orfs downstream of the membrane gene (Quan et al., 2010[121]; Pfefferle et al., 2009[117]).

The families of Vespertilionidae and Molossidae, which are closely related to those of Vespertilionidae, are the most likely to be the progenitors of MERS-CoV. Some examples of these CoVs are those from European Pipistrellus bats, the PML/2011 virus from South African- Neoromicia bats (Ithete et al., 2013[59]; Annan et al., 2013[7]), the CoV from the Spanish *Eptesicus isabellinus* and *Hypsugo savii,* and the sequences from the Thai bat guano (Falcon et al., 2011[44]; Wacharapluesadee et al., 2013[153]). More remote than previously assumed, MERS-CoV was related to CoVs from Ghanaian Nycteris bats (Pfefferle et al., 2009[117]). Only one bat from the Emballonuridae family, *Taphozous perforatus* of Saudi Arabia, has a 203-nucleotide RdRp sequence fragment that was similar to the original MERS-coronavirus strain; EMC/2012 (Su et al., 2016[144]). The higher sequential identity of this specimen has made it easy to identify further CoV genomic areas. However, no more viral sequences could be acquired from this specimen.

It's rare, but not impossible, to discover the closely associated coronaviruses in bat's diverse species (Lau et al., 2012[76]). A lack of more CoV sequencing data and a single genome fragment in the two indistinctly associated bat families i.e., Emballonuridae and Vespertilionidae are needed to support this result. A 202-nucleotide sequence from the *Rhinopoma hardwickii* bat was found to be identical to other β-CoV 1 reference strains obtained in the same study (Memish et al., 2013[96]). The identification of clade A beta coronaviruses in bats will need further individual bats and additional CoV sequencing data, therefore, this is still an unconfirmed hypothesis. High antibody frequencies and titers have been discovered in the instance of camels as putative transitional hosts for MERS-CoV (Perera et al., 2013[115]; Reusken et al., 2013[125]). The only approach to determine the probable bat ancestors of MERS-CoV transmission to human beings through camels is by genomic analysis of the viruses prompting this robust antibody response.

Finally, HCoV-NL63 has no known direct bat ancestor, even though the phylogenetic clade that contains this HCoV is surrounded by other bats' CoVs. According to Huynh et al. (2012[57]), recent HCoV-NL63 growth success on immortalized bat cell lines suggests that the virus and bats may be connected, however, all previous studies have revealed that the bat viruses analyzed are inherently very distant from the HCoV-NL63 (Corman et al., 2013[36]).

So far, we have found nine different coronavirus variants. The sequence similarity with SARS-CoV was found to be much higher than that of MERS-CoV. Though very little information is available, several studies are presently being performed to better recognize the genetic progression of SARS-CoV-2 (Grifoni et al., 2020[47]). As a result, current research suggests that the S protein of SARS-CoV-2 binds to the ACE2 receptor better than SARS-CoV. According to Wan et al. (2020[154]), the six amino acid residues in the S-protein of the SARS-CoV-2 are critical for the virus's attachment to the human ACE2 receptor and host specificity. The specificity and affinity for the human ACE2 receptor, which has a higher degree of similarity with SARS-CoV, are enhanced by the substitution of five of the six amino acid residues in SARS-CoV-2. The core structure and binding motif of the SARS-CoV-2 receptor help to identify and stabilize the human ACE2-receptor structure and attach the virion to it (Shang et al., 2020[135]). 

There are many different receptor-binding patterns in the strains that have been studied, making it easy for mutations to alter the host selectivity and host range (Wu et al., 2012[163]). The RBD of the SARS-CoV-2 confirms the loops in the human ACE2 binding ridge (Shang et al., 2020[135]). The higher specificity of S-protein of SARS-CoV-2 to ACE2 was also claimed by the team of Anderson et al. (2020[6]) and is mainly attributable to the natural assortment in human ACE2 receptors. Human ACE2 binding ridge has been structurally modified via four residues of amino acid sequences Gly-Val-Glu-Gly (the residue positions 482 to 485), resulting in a more compact ridge and improved interaction with the human ACE2 N-terminal helix (Shang et al., 2020[135]; Wu et al., 2012[163]). Proteases such as furin and other enzymes may effectively break the spike protein-S1 and S2 subunits (Huang et al., 2020[56]), dictating viral infectivity and the range of hosts to which it can infect. The SARS-CoV-2 must have this feature. The 12-nucleotide insert that encodes proline (Chan et al., 2008[20]) may have a role in the insertion of three O-linked glycans surrounding the polybasic cleavage site. A considerable increase in pathogenicity was seen when the avian influenza virus hemagglutinin protein was introduced with polybasic cleavage sites (Alexander and Brown, 2009[3]). According to one study, an O-linked glycan may assist to preserve the epitopes of the S-protein of SARS-CoV-2, which are assumed to be protected by the mucin-like domain (Bagdonaite and Wandall, 2018[12]). The structural and genetic evolution of SARS-CoV-2 investigations will help us to better understand viral virulence and transmission, as well as aid in the effective vaccine development.

## Mechanism of Action: Receptors Binding Phenomenon

The core and receptor-binding subdomain of the MERS-CoV-RBD are the most critical elements of the RBD (Wang et al., 2013[157]). It is extremely similar to SARS-CoV RBD in terms of structural similarities when compared to known structures. In part, this structural difference may explain the MERS-CoV and SARS-CoV specificity. Selection is also pressing for structural convergence, in spite of the higher degree of sequence diversity, in the core domain. DPP4-propeller domain RBD preferentially attaches to blades 4 and 5, perhaps due to shape and charge complementarities at the binding interface. Specifically, three positively charged residues on the outer surfaces of blades-4 and 5 (K267) of DPP4 engage with the propeller domain of RBD (E536, D510, D537, and D539). The short helix of DPP4 among blades 4 and 5 docks to the hydrophobic concave surface of RBD due to the charge-charge interactions. RBD-binding structural features are only seen in Blades 4 and 5 of DPP4. MERS-CoV RBD binding site is distant from DPP4's enzymatic site, as is the case with ACE2 binding to SARS-CoV RBD14 in the same way. Because DPP4 enzymatic inhibitors did not prevent MERS-CoV from entering the body in earlier investigations, structural explanations for this result are now available. ACE2 and DPP4, on the other hand, have distinct structural characteristics. These two viruses are anticipated to differ structurally, as well as in terms of the degree of expression and distribution of their genes, which will play a crucial role in determining their cell tropism and pathogenicity in humans. MERS-host CoV's range and cell susceptibility may be defined by the sequence variation in the contact residues of DPP4 from distinct animals (Wang et al., 2013[157]).

For SARS-CoV-2 S receptors, Li et al. determined that the greater affinity of human ACE2 than DPP4 for SARS-CoV-2 S indicated that the former is more significant than the latter. MERS and SARS-CoV-2 were shown to be capable of interacting with DPP4 through the same critical binding residues in their respective interfaces, according to these results. In SARS-CoV-1, E484 and the surrounding residues have been replaced and inserted, resulting in a structural alteration that subsidizes the properties of SARS-CoV-1 spikes to bind to DPP4. As far as we can tell, the binding among DPP4 and SARS-CoV-2-S is very unique. One other animal β-CoV, the pangolin-isolated one, has this potential binding capability simply because it contains these identical key residues in the sequences of spike RBD (Li et al., 2020[86]; Lam et al., 2020[74]). Using the models, we were able to determine the binding potential, interface residues, and structural consistency of the known virus-host interactions in SARS-CoV-S/ACE2, MERS-CoV-S/DPP4, and other strains.

Since DPP4 is the receptor for MERS-CoV, it is more likely to infect a coronavirus. DPP4 was unable to allow the entry of SARS-CoV-2 on its own in non-permissive cells like BHK2 and HeLa (Letko et al., 2020[79]; Hoffmann et al., 2020[54]). Even yet, its role in the entry of SARS-CoV-2 into the host cells relics a mystery at this point. The SARS-CoV-2-S RBD has been revealed to bind to DPP4. There is an abundance of DPP4 in human tissues, a serine protease with multiple functions. In addition to the lower respiratory system, liver, kidney, prostate, and small intestine, DPP4 is present in the placenta, lung fibroblasts, wounded skin and muscle, and central nervous system (Cheng et al., 2019[26]; Serej et al., 2017[133]). It is frequently expressed in activated immune cells i.e., CD4(+), CD8(+), B-cells, natural killer cells, dendritic cells, and macrophages. The involvement of chemokines, cytokines, and peptide hormones in a broad spectrum of inflammatory and immunological illnesses is regulated by this molecule (Song et al., 2019[139]; Shao et al., 2020[136]). DPP4 and ACE2, the primary receptor for SARS-CoV-2, must be studied to recognize the possible functioning of DPP4 in this process.

ACE2 is one of the fundamental regulators of the renin-angiotensin system (RAAS). Comparatively, the ACE2 sequence is similar to the tACE and Drosophila ACE sequences (AnCE). Sequence similarity permitted us to create an ACE2 homology model with less than 0.5 root-mean-square divergences from the aligned AnCE and tACE crystal structures. The catalytic site is located in a deep tunnel on top of molecule, which is an important feature of the model. The positively charged RBD of S-glycoprotein may attach to negatively charged ridges nearby the channel. Researchers have discovered patches of hydrophobic residues on the surface of ACE2 that may help with binding (Xiao et al., 2003)[166]. These verdicts state that the S-glycoprotein of SARS-CoV can attach to ACE2, and might help researchers better understand the structure and function of ACE2.

A series of positively charged residues (i.e., K439, K447, H445, R441, and R444) were identified in the electrostatic analysis of the model. Multiple patches of the hydrophobic residues were found around the positively charged loop region by hydrophobic analysis. S RBD modeling is limited by the lack of template structures with high sequence identity, even when the fragment is quite small. This means that the RBD model may vary substantially or perhaps completely from the actual structure. S1 and S2 units are far more difficult to simulate because of their size. The RBDs of CoVs, for example, should reside in S1 rather than S2 in a recent model of S1 and S2, which indicated probable receptor binding sites in S2 instead of S1. So, we employed the RBD model to illustrate the hydrophobic patches and sheets, probable complementary charged surfaces, as well as a study of the RBD fragment secondary structure, which also indicated a predominance of the sheet (Spiga et al., 2003[140]).

## Computational Study Approaches for Different Viral Strains

Obtaining the genomic sequence of viral strains is a highly dignified procedure that has seen tremendous development because of the advancement in second (network-based) and third (specific molecules) generation DNA sequencing technologies. Quality control is a crucial first step before doing any downstream analytics based on raw sequencing data. A multitude of measurements is used to evaluate readability quality. To begin, the sequencing machine assigns a quality rating to each nucleotide, indicating whether or not the nucleotide was sequenced correctly (Pappas et al., 2021[109]).

Despite the increasing number of receptors, advanced computational research is nearly focused primarily on the very same methods and approaches: generating specific and extremely selective chemicals to block specific proteins ("one molecule, one target" methodology) (Steuten et al., 2021[141]). The structure-based drug design (SBDD) for SARS-CoV has been significantly assisted by the availability of virus-related structures of proteins. Though, effective and safe small-molecule medications are urgently required to diminish the virus spreading (Schütz et al., 2020[131]). Several SBDD pathways have been examined thus far, with the majority of them targeting the structural and non-structural SARS-CoV-2 proteins, i.e., viral replication inhibitions and multiplications (Li et al., 2021[83]).

**Figure 3:**
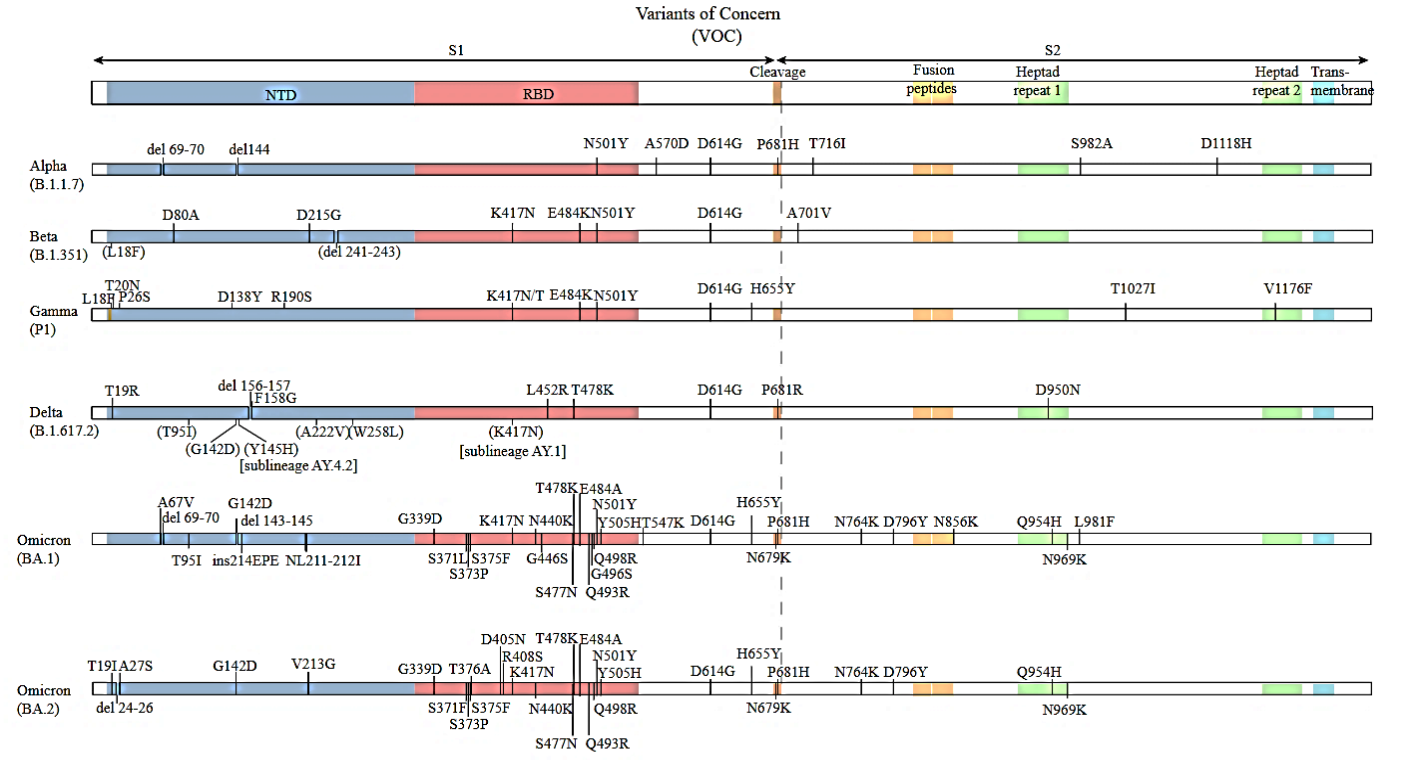
Variants of concerns of SARS-CoV2 overtime (Source: https://viralzone.expasy.org/9556)

Studies have shown that since 2019, many SARS-CoV-2 variants caused by the mutations in the S protein over time have emerged as a variant of concern, as described in Figure 3(Fig. 3). All variants of the SARS-CoV-2 share a set of traits, according to a basic structural analysis (SIB Swiss Institute of Bioinformatics, 2022[137]). Although Wuhan-Hu-1 has 1273 amino acids; Delta variation has 1271, and the Omicron variant has 1270, both contain a few fewer residues than that of the wild-type owing to structural loss (Kumar et al, 2022[73]). When the amino acid compositions of the Omicron and Delta variants are particularly in comparison, the following amino acid constituents substantially increase: arginine (Arg), lysine (Lys), aspartic acid (Asp), and glutamic acid (Glu), insinuating that the Omicron virus may have more charged residues that subsidize to the salt bridge configuration and are largely exposed (Yang et al., 2021[170]). 

Researchers detected the majority of these non-synonymous changes in sequences of spike proteins. Notably, some deletions and insertions discovered in the consecutive bases of Omicron have an impact on the proteins encoded (Parvez et al., 2022[110]) (Table 1(Tab. 1); Reference in Table 1: Parvez et al., 2022[110]). Because of the substantial genetic variations in the RBD of SARS-CoV-2, computational studies revealed that the Omicron variant had a high binding affinity towards the human ACE2 than the Delta variant, indicating a greater possibility of the transmission (Kumar et al., 2022[73]). 

The SARS-CoV-2 structural proteins have fascinating immunostimulatory features and they are the subject of multiple different investigation approaches focused on generating effective vaccines and/or improving sensitive and specific diagnostic models based on formation of antigen-antibody complex, including immunoinformatic strategies (Tilocca et al., 2020[149]). Prior investigations on the vaccine platforms targeting MERS-CoV and SARS-CoV-1 revealed that the RBD domain of S-protein on the viral membrane is a prime target in the development of vaccines as it lowers the human immune potentiation and creates coronavirus neutralization antibodies (Jiang et al., 2005[61]; Kangabam et al., 2021[64]). Furthermore, sequence information, as well as the accessibility of crystalline structures in RCSB PDB of the SARS-CoV-1 (PDB ID: 2GHV), and S-proteins of SARS-CoV-2 complexed with the ACE2 receptors (having PDB ID: 6M0J); that acknowledged the preserved residues which are responsible for the host receptor-binding in SARS-CoV-2, and based upon comparative similarities of sequence-structure to the SARS-CoV (Wrapp et al., 2020[161]; Kangabam et al., 2021[64]). 

SARS-CoV-2 shares about 79 % sequence similarity with SARS-CoV-1 and around 50 % with MERS-CoV at the whole-genome level. Despite their low levels of sequence conservation, CoVs share critical genomic targets (Muratov et al., 2021[102]). Determined by the relative time, the RelTime approach revealed that Neo-CoV had been the oldest, whereas SARS-CoV-1 and MERS-CoV belonged to the equivalent time. Furthermore, phylogenetic analyses of homologous proteins based on MUSCLE and CLUSTALW alignment techniques have demonstrated that merging MERS-CoV and Neo-CoV proteins in the similar clades imply that they have been nearest on the grounds of all strategies employed (Hassan et al., 2020[53]). 

Researchers claimed that Neo-CoV differs from the MERS-CoV by one amino acid exchange only (0.3 %) in translated 816 nucleotide RdRp gene fragment and by 10.9 % amino acid sequence distance in the gene, that encrypts the glycoproteins which are responsible for the attachment of CoV and cellular entry. These findings indicate that Neo-CoV was far more closely linked to MERS-CoV than any anther virus (Calisher et al., 2006[16]). According to Corman and colleagues, 85 % of the Neo-CoV genome appeared chromosomally identical to the MERS-CoV, indicating that perhaps the two viruses conveyed essential characteristics of genome organization and hence adhered to almost the same viral lineage. Because the NeoCoV spike genome contains a hereditarily divergent S1 component, intra-spike recombination events may have contributed a substantial role in the MERS-CoV evolution (Corman et al., 2014[35]).

## Pathogenesis, Mode of Transmission, and the Severity of Several Diseases

### Pathogenesis of SARS-CoV infection

The pathways lying behind the highly intense SARS-CoV pathogenesis are not yet entirely apprehended (Liu et al., 2020[88]). Severe pulmonary impairment in SARS-CoV-infected cases seems to be linked to initially increased viral titers (Peiris et al., 2003[113]), elevated pulmonary monocyte, macrophage, and neutrophil infiltration (Nicholls et al., 2003[106]), and augmented levels of serum pro-inflammatory chemokines and cytokines (Wong et al., 2004[160]). Hence, the medical worsening of the SARS-CoV infection might come from a collection of direct viral-driven cytopathic outcomes and immunopathology caused by hypercytokinemia or a cytokine storm. Reports of the modifications in cytokine or chemokine behaviors in the course of the SARS-CoV infection uncovered elevated levels of traveling cytokines, like tumor necrosis factor α (TNF-α), and interleukins (IL-6 and IL-8), imparted to the unfortunate prognosis in the SARS-CoV infections (Kong et al., 2009[70]). 

Elevated serum levels of pro-inflammatory cytokines such as IL-1, IL-6, IL-12, Interferon γ [IFN-γ], chemokines (CCL2, CXCL9, and CXCL10), and transforming growth factor-β were detected in cases infected with SARS with intense illness as compared to persons with simple or mild SARS. Besides, the rapid initiation of CXCL10 and IL-2, and the resultant hyper-expression of IL-6 with a coexisting deficiency in IL-10 expression are believed to add to the immuno-pathological activities related to pulmonary impairment in the course of SARS-CoV infection (Chien et al., 2006[27]). Likewise, vigorous, and continual production of IFN-α, -γ, and IFN-stimulated genes (ISGs) come with early SARS abnormalities (Cameron et al., 2007[17]). It has well been proved that SARS-CoV infections lead to a deferred production of type I IFN. The deferred-kind I IFN signaling that comes with vigorous viral replication, was observed to boost the aggregation of pathogenic inflammatory monocyte or macrophages, leading to raised pulmonary cytokine or chemokine levels, vascular leak, and damaged viral-unique T cell reactions (Channappanavar et al., 2016[23]). 

### Pathogenesis of MERS-CoV infections

DPP4, the start of MERS-CoV receptors, is broadly produced on the renal, pulmonary, gastrointestinal, and prostatic epithelial cells and triggered leukocytes (Widagdo et al., 2016[159]), declaring that the extent of MERS-CoV tissue tropism is wider than that of whatever another coronavirus (Song et al., 2019[139]). MERS-CoV was observed to infect more human immune cells, considering dendritic cells (DC) (Chu et al., 2014[31]), macrophages (Zhou et al., 2014[178]), and T cells (Chu et al., 2016[30]). MERS-CoV infections of macrophages and DCs lead to vigorous and continuous expression of pro-inflammatory cytokines and chemokines like TNF-α, IL-6, CXCL-10, CCL-2, CCL-3, CCL-5, and IL-8 (Chu et al., 2014[31]). Pro-inflammatory and immune-drawing capabilities of such cytokines are believed to result in (or minimally impart to) immune cell penetration into the lower respiratory tract of infected cases and determined intense inflammation and tissue impairment (Zhou et al., 2014[178]). 

MERS-CoV infections of T cells cause programmed cell death, mediated through a collection of internal and external programmed cell death mechanisms. Via such a pathway, MERS-CoV avoids the T cell reaction in the peripheral blood and lymphoid organs which might boost viral transmission and intense immuno-pathogenesis (Chu et al., 2016[30]). It has been described that MERS-CoV may likewise stimulate both renal and pulmonary programmed cell death via upregulation of fibroblast growth factor 2 (FGF2) and Smad7 production (Yeung et al., 2016[172]).

### Mode of transmission 

Respiratory viral infections such as SARS-CoV-1, MERS-CoV, and COVID-19 always spread through direct contact between individuals via airborne particles and respiratory droplets (Peeri et al., 2020[111]). The aerosol spread was described to have a crucial function in the transmission of MERS-CoV, SARS-CoV-1, and SARS-CoV-2 viruses (Nardell and Nathavitharana, 2020[103]). Aerosols are droplets smaller than five µm, which could stay suspended in the air for prolonged time (Tellier, 2009[147]). In laboratory conditions, the active aerosol efficiency of SARS-CoV-2 excelled over that of MERS-CoV, SARS-CoV-1, and respirable SARS-CoV-2 aerosols held infectivity and particle state, reaching sixteen hours (Doremalen et al., 2020[40]). However, sneezing and coughing generate more aerosols per breathing maneuver than natural breathing. Still, natural breathing could produce aerosols (Wu et al., 2021[165]), showing the spreadability of asymptomatic and mildly infected individuals. The incubation period, described as the number of days from exposure to the virus till starting of symptoms, is alike between the three hCoVs (Table 2(Tab. 2); References in Table 2: Chan et al., 2015[21]; Cui et al., 2019[37]; Jiang et al., 2020[62]; Li et al., 2005[85]; Wrapp et al., 2020[161]; Yin et al., 2018[173]; Zhou et al., 2020[179]).

### Severity of several diseases

#### Kidney

MERS and SARS have been linked to acute kidney injury (AKI), which might originate from the viral tropism of the kidneys and secondary impairment because of systemic inflammation and hypotension (Lombardi et al., 2021[89]). In a retro study of 536 infected cases with SARS, 6.7 % showed AKI, and 91.7 % of them consequently passed away (Chu et al., 2005[32]). 

MERS-CoV infection commonly makes intense extra-pulmonary organ impairment, and most cases develop AKI and thrombopenia (Arabi et al., 2014[9]). In other retro studies with thirty infected cases with MERS, eight (26.7 %) cases suffered AKI, and 15 (50 %) presented albuminuria (Cha et al., 2015[19]). In such a study, aged cases had a greater incidence of AKI. The precise pathway of kidney impairment is not well realized; reports have proved virus tropism for renal cells *ex vivo*, declaring MERS-CoV may stimulate programmed renal cell death (Yeung et al., 2016[172]; Eckerle et al., 2013[41]). 

Data from 333 cases institutionalized with pneumonia from COVID-19 were studied and proved that 75.4 % presented renal impairment (Pei et al., 2020[112]). Renal abnormalities in twenty-six autopsies of COVID-19 cases, from them nine cases, had medical clues of the renal implement. The highly considerable findings were proximal tubule lesion, loss of brush border, and necrosis (Su et al., 2020[143]).

#### Endocrinal system

A study showed that cases recovered from SARS have presented problems in fat and sugar metabolism, with raised phosphatidylinositol (PI) and lysophosphatidylinositol (LPI) levels (Wu et al., 2017[164]). Since such fats are included in insulin metabolism, such modifications might lead to issues in sugar metabolism, elevating glucose tolerance, and insulin production.

A second study detected that SARS accompanying coronavirus could cause renal, cardiac, pulmonary, and pancreatic impairment, probably because of elevated production of ACE receptors. In such a study, over 50 % of the cases went diabetic during hospital admission because of the SARS-CoV infection. They recommended coronavirus may get into the pancreas through the ACE2 receptor, leading to acute islet cell impairment and therefore cutting down insulin production, causing transient type-II diabetes mellitus and acute hyperglycemia (Yang et al., 2010[168]). SARS has also been involved in hypocortisolism and hypothyroidism in about sixty-one recoverees of the viral infection. They concluded it could be because of a transient hypophysitis or direct hypothalamic outcome resulting from the virus (Leow et al., 2005[78]). 

A study reported a patient diagnosed with intense acute pancreatitis who had COVID-19 (Aloysius et al., 2020[5]). A second study described acute pancreatitis related to having SARS-CoV-2, where two cases showed raised amylase in plasma and radiological features of acute pancreatitis. The researchers supposed that direct viral penetration could be related to enzymatic stimulation, auto-digestion, complement system stimulation, micro-circulation disruption, and necrosis (Hadi et al., 2020[51]). The researcher of that paper describes a hospitalized patient diagnosed with COVID-19 who showed ground-glass opacities on chest CT, besides accompanying abdominal pain and elevated levels of amylase, and was subsequently diagnosed with acute pancreatitis.

However, the existing data are limited, as cases with adrenal impairment might be in more danger of clinical complications and death from COVID-19. Ill-fed cases diagnosed with COVID-19 infection might likewise be in more danger of malnutrition because of the raised inflammatory body reaction and higher nutritive demands (Puig-Domingo et al., 2020[120]). 

#### Liver 

Reports have described a diversity of hepatic problems because of SARS and MERS, regarding mild to moderately raised transaminases, hypo-albuminemia, mild steatosis, congestion, and necrosis. Percutaneous hepatic biopsies in three cases presented liver cell infection by SARS-CoV and higher transaminases. The researchers detected a noticeable aggregation of cells in mitosis and apoptosis (Kukla et al., 2020[72]). The mechanism of invasion is via ACE2 receptors, which are extravagantly produced on the endothelia of hepatocytes (Xu et al., 2020[167]). 

As detected in SARS-infected patients, MERS leads to mild hepatic inflammation. Yet, the pathway of getting into the cell is varied via other forms of cell receptors (DDP-4) that, as well, are increasingly produced in liver cells (Raj et al., 2013[123]). Hepatic dysfunction linked to COVID-19 has likewise been reported (Fan et al., 2020[45]). 

#### Neuromuscular system

Coronaviruses could attack the neural system leading to a broad extent of medical neural complications that could be reached in many ways, covering trans-synaptic transmission, straight penetration through the smelling neurons, endothelium, or movement through the blood-brain barrier (BBB) (Zubair et al., 2020[180]). A study reported neuromuscular symptoms in SARS-diagnosed cases: two cases showed motor-predominant peripheral neural conditions, one had myopathy, and another had both myopathy and neuropathy (Tsai et al., 2004[150]). Another study described three SARS cases that had rhabdomyolysis during therapy. Each administered succinylcholine for muscle blockade during staying in the ICU could have been affected as a contributing factor (Wang et al., 2003[155]). Peripheral neuropathy has besides been reported in SARS cases, and critical illness polyneuropathy might be thought of as the reason (Chao et al., 2003[24]). 

Cerebral infarct might be related to SARS, considering a study of 206 cases, of whom five had ischemic strokes, yet only two had earlier risk elements (Umapathi et al., 2004[151]). The origin is unidentified, however, prescribing intravenous immunoglobulin could be linked to such a result (Dalakas et al., 2003[38]). Other speculated pathways might be vasculitis or hypercoagulable condition (Ding et al., 2003[39]).

Considering the ongoing COVID-19 pandemic, different pieces of research report neural complications. Anosmia, headache, and hypogeusia are often initial symptoms of CoV infections. The cerebral ischemia happens likely because of virus penetration of the endothelium leading to coagulopathy, vasculitis, and thrombosis. Seizures, myelitis, encephalopathy, meningitis, Guillain-Barré, and Miller-Fisher have likewise been reported (Román et al., 2020[126]). Penetration of the medullary cardiorespiratory center with SARS-CoV-2 could be causative of refractory respiratory impairment in critical cases (Montalvan et al., 2020[99]). Face palsy could likewise happen after SARS-CoV-2 infections (Caamaño and Beato, 2020[14]). A study described six COVID-19-diagnosed cases who had a stroke during therapy, yet all cases, except one, had accompanying vascular risk elements. The result was misfortunate, with five cases passing away and one staying severe neuronally impacted (Morassi et al., 2020[100]). 

COVID-19-infected cases have likewise shown symptoms of cranial peripheral neuropathy, such as internuclear ophthalmo-paresis and oculomotor paralysis (Gutiérrez-Ortiz et al., 2020[50]). Hence, it is crucial to be alert that some neuronal symptoms reported in cases with such viral infections are not unique and have substantial convergence with other intense infections and mostly there is no explicit verification of virus infection in the cerebrospinal fluid of SARS-CoV-2 cases (Needham et al., 2020[105]). 

Late imaging studies supplied growing proof of central nervous system conditions in cases infected with SARS-CoV-2, especially white matter signal modifications (Kreme et al., 2020[71]), which might include the corpus callosum (Sachs et al., 2020[128]), and likewise, various instances of acute necrotizing encephalopathy (Poyiadji et al., 2020[119]), acute ischemic infarctions (Mahammedi et al., 2020[93]), micro-bleeding (Radmanesh et al., 2020[122]), basal ganglia conditions (Chougar et al., 2020[29]), encephalomyelitis, meningitis, cranial and spinal nerve root conditions (Klironomos et al., 2020[68]), bringing out the value of neuronal surveillance, particularly in critical cases (Katal et al., 2021[66]).

#### Cardiovascular system

There are scarce studies considering cardiovascular conditions from SARS-CoV-1 infections, with few reported data of cases with acute coronary syndrome, transient diastolic impairment, myocardial infarct, hypotension, cardiac arrhythmia, transient megalo-cardia, and a postmortem examination that presented thromboembolic condition (Madjid et al., 2020[92]; Peiris et al., 2003[113]; Yu et al., 2006[174]; Li et al., 2003[84]). Cardiovascular conditions in MERS likewise have scarce data. Most of the produced studies are from case reports or regarding the pervasiveness of comorbidities in infected cases (Badawi et al., 2016[11]). Elevated troponin and radiology of myocardial inflammation were reported in a study (Alhogbani, 2016[4]).

COVID-19-diagnosed cases have been described to show cardiac arrhythmia and myocardial inflammation (Wang et al., 2020[156]). In a study, 5 patients developed a cardiac impairment (Huang et al., 2020[56]). In a studied 187 COVID-19-diagnosed cases, 52 (27.8 %) developed cardiac muscle impairment as shown through raised levels of troponin, and the cardiac muscle impairment was importantly related to deadly results (Guo et al., 2020[49]). Acute pericarditis was reported in a COVID-19-diagnosed case that showed pleuritic symptoms and pericardiac effusion, which was declared following colchicine therapy. The pathway of cardiac conditions is not lucid; however, theories point to potential direct viral injury and auxiliary inflammatory procedures (Clerkin et al., 2020[34]).

Cases of COVID-19 have more clotting conditions. Research has shown the involvement of the inflammatory reaction and stimulation of the clotting cascade (Levi and Thachil, 2020[80]). These theories have been sustained by many documented cases of thromboembolic complications following cytokine storms, without having any risk elements for thromboembolism (Griffin et al., 2020[46]).

Late studies have contributed to the knowledge of cardiac conditions, considering dropping cardiac muscle work, myocardial inflammation, pericarditis, artery thrombosis, pericardiac effusions, and coronary artery aneurysms (Hameed et al., 2021[52]). Pneumonic thromboembolism is especially prevalent in badly sick cases and includes principally segmental and sub-segmental arteries of pulmonic segments impacted by consolidation, increasing fears of inflammatory and hyper-coagulability elements lending to its pathological process (Cavagna et al., 2020[18]). Many cases might show cardiac conditions without signs or symptoms of interstitial pneumonia, as described in a case subsequently diagnosed with myopericarditis (Inciardi et al., 2020[58]).

#### Pregnancy and perinatal problems

Coronaviruses might be related to perinatal complications. Lately, an orderly review of the gestational-associate problems from MERS-CoV, SARS-CoV-1, and SARS-CoV-2 presented an elevated pervasiveness of pre-term births and abortion, coupled with fetal distress, and the need for ICU admission (Mascio et al., 2020[95]).

## Different Preventive Measures, Control, and Treatment Aspects of Viral Infections

### Different preventive measures and control

Worldwide researchers are acting on discovering methods to handle the coronavirus pandemic. The first direction is to lessen the coronavirus-associated fatality rate (Sarangi et al., 2022[129]). The extension of the illness could be decreased via the enforcement of medical care and community guidelines that are frequently revised by the health authorities. Hence, preventive activities, keeping off hospital-acquired infections, and decreasing coronavirus- and worry-related psychological health issues are evenly crucial (Adhikari et al., 2020[2]). 

#### Physical distancing 

Carrying out assertive physical distancing activities to decrease direct interaction among individuals is an effective and potent scheme to diminish viral spread among people, and to cut down the disease-related death rate during the pandemic. Absolute containment was applied in various states globally and has presented an advantageous effect, importantly trimming the growth in the figure of cases. Social distancing activities such as isolating infected persons, quarantining close contacts, possibilities for individuals to work virtually, shutting down schools, and forbidding big assemblages have likewise been potent. The WHO urges maintaining a lower limit spacing of 1 m (3.28 ft) among persons to keep the infection transmission via infected respiratory droplets (Varghese and John, 2020[152]). 

#### Personal protection measures 

Personal protective activities are an inherent component of preventing and controlling infection and are indicative of the tone of community engagement. It includes activities on the far side of governmental plans of action, which could assist in making a healthy time to come and palliate the existing condition. Proper hand hygiene, kept via rinsing hands with water and soap, could highly trim the prevalence of infection and the transmission of the disease. If an individual shows respiratory symptoms, the utilization of a medical mask is urged. Nevertheless, putting on rubber gloves in the community is deterred and does not supersede the demand for hand hygiene. One should have disinfectants and cleaning agents when often encountering dirty artifacts (Varghese and John, 2020[152]). Putting on a face mask and making proper hand hygiene could assist in preventing coronavirus infection and decrease the spread of other respiratory infectious diseases (Chiu et al., 2020[28]; Chan, 2020[22]). 

#### Isolation at home

Cases suspected of coronavirus infection are evaluated via a healthcare provider to assess if the case could be cared for at home; cases could be supplied with symptomatic treatment. Cases are counseled to keep apart in a well-aired area, which is not used by other family members. Mobility to spots external to this area should be limited, and cases should be limited to the dwelling to decrease the hazard of spread. One caregiver, commonly one of the case's households, should be charged as the case's attendant. The caregiver should keep proper spacing in helping the case with whatever demands. Visitants must not be permitted to get into these areas until the case completely improves. Every family member should keep off direct interaction with the case. Family members should be suspected of coronavirus, and should, hence, follow quarantine programs. The case's health condition should be supervised through an entire incubation period (Varghese and John, 2020[152]).

#### Caring for susceptible groups

A captious aspect of coronavirus is the disproportionately greater death rate found among persons of old age, compared with young ones or children. Because youngsters might frequently be a source of disease spread, they are necessitated to restrict their contact with seniors. Help in food market purchasing and the delivery of food, medications, and necessary services could mostly decrease unneeded danger for the susceptible groups. These wisely bedded physical distancing interventions might be a more satisfactory and long-term resolution to the circulating pandemic (Varghese et al., 2020[152]).

### Treatment aspects of viral infections

#### Obstructing de novo infection

A major pathway of action for antivirals is obstructing *de novo* infection. The obstruction could be generated via agents such as human-neutralizing antibodies supplied as monoclonal antibodies or in convalescent plasma, inhibitors of virus entry, and/or antibody elevation through immunization (Tian et al., 2020[148]; Wang et al., 2020[156]). For instance, a SARS-CoV-unique human monoclonal antibody, bamlanivimab had emergency utilization permission through the US Food and Drug Administration (USFDA) for the management of SARS-CoV-2.

Broader medicine effectiveness and sooner management are linked to finer results: Based on one study, the area under the curve (AUC) was decreased by 73 % and 74 % of target cells persisted uninfected after the course of infection, when management was started 1 day following symptom attack and the antiviral effectivity was 90 %. Exceedingly early management is cardinal for finer results during antiviral agents. However, administering a medication that obstructs infection with 95 % effectiveness started 4 days following the symptom attack, the AUC was decreased by only 14 %, and only 2 % of cells are uninfected. This comes since only a small part of target cells stay uninfected following the virus load peak. Similar forms for MERS-CoV and SARS-CoV are detected, however starting the therapy a couple of days following the symptom attack might be effective. The virus load peak was found to take place subsequently for SARS-CoV and MERS-CoV than for SARS-CoV-2. Therefore, if therapy is started 4 days following symptom start, that is earlier virus load peak for the two viruses, better results could be anticipated (Kim et al., 2021[67]).

#### Obstructing viral production

A lot of antiviral agents suppress intracellular viral replication. HIV protease inhibitors (lopinavir or ritonavir), anti-*Ebola* virus disease candidates (remdesivir), and other nucleoside analogs, besides interferon, can inhibit replication of SARS-CoV-2. Like the insights from agents obstructing *de novo* infection, broader effectiveness and initial therapy are related to improved results. Based on one study, the AUC was decreased by 76 %, and 36 % of the target cells stayed uninfected following the infection if therapy was started 1 day following the symptom attack and the antiviral effectivity was 90 %. However, if therapy is initiated following the virus load peak, better results could not be anticipated even with a 100 % suppression rate. Related forms were detected for MERS-CoV and SARS-CoV. Nevertheless, as 4 days following symptom start is yet earlier than the viral load peak for such two viruses, a significant advance in the results is anticipated with therapy started 4 days following symptom attack for such two viruses (Yao et al., 2020[171]; Lu, 2020[90]; Kim et al., 2021[67]).

#### Boosting cytotoxicity

Another potential antiviral pathway is to boost cytotoxic effects, which may be caused via evoking adaptive immunity, involving reactions induced through cytotoxic T lymphocytes, natural killer cells, immunotherapy, or immunization. However, the outcome might not be direct. To be coherent with the other conditions of medicine effect covered earlier, where it is expected that the agent acts instantly following administration, such as a virus-unique monoclonal antibody combined with a toxin, as applied in malignant tumor treatment (Hoffmann et al., 2020[54]), or a non-neutralizing virus-unique monoclonal antibody, which might stimulate the death of infected cells via complement-induced lysis or antibody-reliant cells cytotoxicity. A neutralizing antibody with such actions might be seen as the same as the combination treatment that is covered beneath. Compared with the other two treatment pathways of effect (obstruction of *de novo* infection and viral production), the initiation of cytotoxicity immediately takes away infected cells, which generate viruses, and hence it improves the rate of virus load decay. Following the virus peak, target cells are consumed, and cytotoxicity-generating treatment results in observably higher speed decay in virus load.

To assess the outcome of therapy in boosting cytotoxicity started following the virus load peak, they analyzed the outcome of a 50 % effectual therapy started at 1 day and 13 days following symptoms attack on the entirely three coronaviruses. The treatment started 1 day and slowed the time of the virus load peak, especially for SARS-CoV and MERS-CoV. When the therapy was started at 13 days that is following the virus load peak, the virus load decreased quickly compared with therapy starting at 1 day, since a couple of target cells stayed, and hence rising infection was restricted. The investigation of the therapeutic outcome of agents with three several ways of action uncovered that the therapeutic scheme must be diverse for every kind of agent. For instance, applying agents that obstruct *de novo* infection or viral production could keep off significant target cell decrease if started before the virus load peak. Using an agent which boosts cytotoxicity is a little time-sensitive, and therapy started following the virus peak could decrease the AUC. Such insights imply the prospect of a synergetic outcome of adding agents with ways of action (Kim et al., 2021[67]).

#### Combination treatment

Overall, combinations of antiviral treatments are seen as desirable when they synergistically intensify the antiviral outcomes, cut down the required case-by-case medicine dose, and trim the adverse events compared with a single treatment (Ohashi et al., 2020[107]; Martyushev et al., 2016[94]; Koizumi et al., 2017[69]; Laskey and Siliciano, 2014[75]). 

A study found that the three potential therapeutic combinations are obstructing *de novo* infection and viral production, obstructing viral production and boosting cytotoxicity, and obstructing *de novo* infection and boosting cytotoxicity. Entirely, the three combination treatments enhanced the antivirus outcomes compared to the related single agent. The addition of agents with defined mechanisms of action, particularly an agent that increases the cytotoxicity of these agents, lowered the AUC and protected target cells from infection. With a single agent of a 50 % antivirus outcome started 1 day following the symptom attack, the AUC was decreased by 13 %, 44 %, and 54 % with the agents obstructing *de novo* infection, obstructing viral production and boosting cytotoxicity, respectively, where the AUC was decreased by 58 % or more using combination treatments. Besides, adding an agent boosting cytotoxicity with one of the other two kinds of agents made up for the imperfection of each one. Additionally, they expected no distinct outcome from the agents obstructing *de novo* infection or viral production if started following the virus load peak (Kim et al., 2021[67]).

Biologically, boosting cytotoxicity is defined from the other two pathways. Both obstructing *de novo* infection and viral production restrict current *de novo* infection, where boosting cytotoxicity raises viral and infected cell discharge irrespective of target cell availability. A neutralizing antibody with strong effector actions that stimulated infected cell mortality could be an ample treatment choice, as it stimulates two ways of action in one molecule. Antibodies of such kinds are being researched for HIV (Asokan et al., 2020[10]; Wang et al., 2020[156]). SARS-CoV-2 neutralizing antibodies are likewise in clinical investigations and their effector actions in supplying prophylactic action are studied. One study also found that immunomodulation with antiviral agents can reduce viral load as a side effect, even when therapy is started after viral load has peaked. They observed similar patterns for both SARS-CoV and MERS-CoV (Schäfer et al., 2021[130]).

## Conclusion

SARS-CoV, MERS-CoV, SARS-CoV-2, and influenza A viruses are the most common pathogens, that primarily attack the human respiratory system. Their infections may cause diseases ranging from mild respiratory illness to acute pneumonia and even respiratory failure. The recent epidemic of SARS-CoV-2 infection has instigated a worldwide crisis in the epidemiology and medical systems. In addition to a brief overview of the structural features and morphological characteristics, sources of the virus's origin, infection mechanism, computational study approaches, pathogenesis, and possible therapeutic approaches, we summarized and compared the immune responses to SARS-CoV, MERS-CoV, and SARS-CoV-2. This may guide using immune therapy as a combined treatment to prevent the patients from developing the severe respiratory syndrome and largely reduce the complications.

## Declaration

### Conflict of interest

The authors declare no conflict of interest in this article.

### Acknowledgments

Authors are thankful to their organizations for their support.

### Author contributions

All authors listed have made a substantial, direct, and intellectual contribution to the work, and approved it for publication.

## Figures and Tables

**Table 1:**
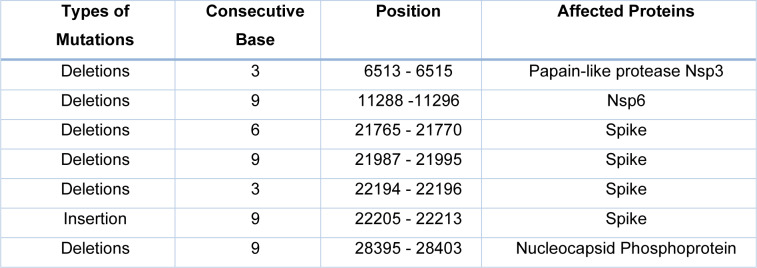
Consecutive mutations in omicron variant (Data source: Parvez et al., 2022)

**Table 2:**
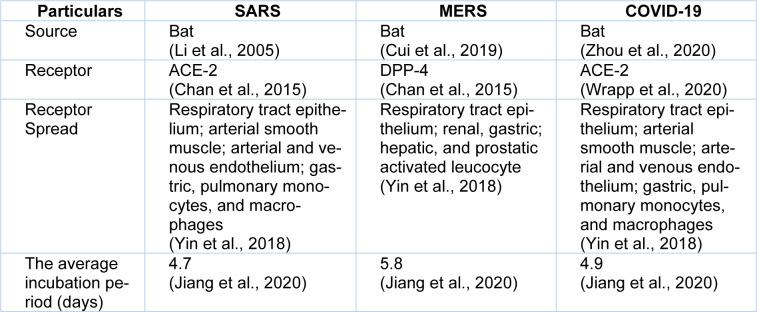
Comparability of some epidemiological characteristics of SARS-CoV-1, MERS-CoV, and COVID-19
